# Determinants of implementation of continuous glucose monitoring for patients with Insulin-Treated type 2 diabetes: a national survey of primary care providers

**DOI:** 10.1186/s12875-025-02764-7

**Published:** 2025-03-08

**Authors:** Varsha G. Vimalananda, Ben Kragen, Alison J. Leibowitz, Shirley Qian, Jolie Wormwood, Amy M. Linsky, Patricia Underwood, Paul R. Conlin, Bo Kim

**Affiliations:** 1 Center for Health Optimization and Implementation Research, VA Bedford Healthcare System, Bedford, MA USA; 2 Section of Endocrinology, Diabetes, Metabolism and Weight Management, Boston University Chobanian and Avedisian School of Medicine, Boston, MA USA; 3 Department of Psychology, University of New Hampshire, Durham, NH USA; 4 Center for Health Optimization and Implementation Research, VA Boston Healthcare System, Boston, MA USA; 5 Section of General Internal Medicine, Department of Medicine, Boston University Chobanian & Avedisian School of Medicine, Boston, MA USA; 6 New England Geriatric Research Education and Clinical Center, VA Boston Healthcare System, Boston, MA USA; 7 Veteran Affairs Boston Healthcare System, Boston, MA USA; 8 William F. Connell School of Nursing, Boston College, Boston, MA USA; 9 Harvard Medical School, Boston, MA USA; 10 Department of Psychiatry, Harvard Medical School, Boston, MA USA

**Keywords:** Continuous glucose monitoring, Diabetes mellitus, Type 2, Primary health care, Implementation science, Surveys

## Abstract

**Objectives:**

To identify determinants of continuous glucose monitoring (CGM) implementation from primary care providers’ (PCPs’) perspectives and examine the associations of these determinants with both PCP intent to discuss CGM with eligible patients and facility-level uptake of CGM.

**Study design:**

Cross-sectional survey.

**Methods:**

A survey about CGM implementation for patients with type 2 diabetes on insulin was distributed to all PCPs in the Department of Veterans Affairs (VA) health system from October 2023-April 2024. Multi-item scales measured perceived clinical benefits of CGM, workload capacity, knowledge about CGM, access to CGM resources, and support from leadership and other services. Responses were on a 5-point Likert scale from “Strongly Disagree” to “Strongly Agree”. An item asked about likelihood of initiating discussions about starting CGM. Facility-level uptake was measured using VA administrative data. Multivariable regression models assessed the relationship between determinants of CGM implementation and both PCP intent to discuss CGM and facility-level uptake.

**Results:**

Of 1373 respondents, most perceived clinical benefits of CGM (79% “Agree” + “Strongly Agree”). Very few indicated sufficient access to resources (8%) and support from leadership & other services (5%). After adjustment for respondent characteristics, the scale most strongly associated with PCP intent to discuss CGM was PCP Knowledge About CGM (B = 0.54, *P* <.001). Facility uptake of CGM was associated with Clinical Benefits of CGM (B = 0.10, *P* =.026) and Support from Leadership & Other Services (B = 0.18, *P* <.001).

**Conclusions:**

PCPs perceive benefits to CGM but lack sufficient knowledge, resources, and workload capacity to manage it alone. PCP education about CGM use and interprofessional support for uptake may increase the likelihood that eligible patients use CGM.

**Supplementary Information:**

The online version contains supplementary material available at 10.1186/s12875-025-02764-7.

Managing blood glucose to maximize the time in target range is required to prevent major complications of diabetes, but fewer than 60% of adults with diabetes achieve recommended glucose targets [1]. Continuous glucose monitoring (CGM) is a significant advance for diabetes management that, while standard of care for management of type 1 diabetes mellitus, is increasingly used for insulin-treated patients with type 2 diabetes mellitus [2–4]. Randomized trials in T2D of CGM versus self-monitoring of blood glucose with fingersticks show improved A1C, hypoglycemia, and diabetes-related distress [5–11]. CGM can reduce A1C by about − 0.4% vs. usual care [8, 12], which is clinically meaningful [13]. Observational studies of CGM in T2D find decreases in emergency room visits [12, 14] and hospitalizations [12, 14, 15]. Observational research in VA is consistent with these findings, with more CGM users achieving glycemic control and a 10% reduction in all-cause hospitalization [15, 16]. 

CGM is increasingly covered by payers for patients with type 2 diabetes on insulin. This expanded eligibility warrants consideration of how the technology will be supplied and supported for the large majority of patients with type 2 diabetes whose condition is managed in primary care. Historically, CGM was a tool utilized by endocrine specialty clinicians trained and experienced in managing type 1 diabetes and complex type 2 diabetes using CGM. Given the narrow indications for CGM until relatively recently, primary care staff have not needed that background. However, implementing CGM without prior experience can be a challenge. Clinical staff must be able to determine appropriate patient eligibility; ensure patients and caregivers receive training and ongoing support; and access, interpret, and act on data. Primary care clinics may have difficulty integrating CGM into care due to few pre-existing resources to support implementation of CGM prescribing, patient education, data monitoring, and ongoing management [2, 4, 17–19]. Specific barriers to CGM implementation may include lack of training, challenges with workflow integration, financial constraints, and challenges with electronic health record (EHR) integration [20–24]. Without adequate support for CGM implementation, uptake in primary care may be hindered and patients who would otherwise benefit may not be offered the technology.

We conducted a survey study of primary care providers (PCPs) to understand which factors are most strongly associated with CGM implementation as measured in two ways: (1) PCPs’ self-reported likelihood of initiating discussions about starting CGM with potentially eligible patients, and (2) facility-level uptake of CGM. Our goal was to identify which determinants to prioritize for improvement efforts focused on supporting CGM use for patients with type 2 diabetes on insulin.

## Methods

### Study setting

The VA is the largest integrated health system in the United States, serving over 9.1 million Veterans annually across 170 medical centers and 1,193 outpatient sites of care [25]. In July 2023, VA expanded CGM eligibility from patients on complex insulin regimens under VA endocrine specialty care to the much larger pool of patients with type 2 diabetes on any insulin regimen, who are often managed in VA primary care. The survey was administered to evaluate PCP perspectives under the new conditions of being able to prescribe CGM.

### Survey development

We created a questionnaire to assess PCPs’ perspectives on what was needed to support CGM implementation for patients with insulin-treated type 2 diabetes whose condition was not being managed in endocrine specialty care. We used the Consolidated Framework for Implementation Research (CFIR) [26] to guide survey development. CFIR is a widely recognized framework for understanding multilevel factors associated with health services delivery. CFIR includes 48 constructs across five domains, four of which were relevant to this study: Innovation, Individuals, Inner Setting, and Outer Setting.

For each CFIR construct, two members of the research team determined one or more ways that the construct could be understood as a factor influencing implementation of CGM. The research team included diabetologists with expertise in clinical care and leadership, a primary care provider, and health services researchers with various expertise including implementation science. We identified 52 determinants in language specific to CGM implementation. We then conducted a multi-voting exercise, during which each research team member was asked to vote on the 20 factors most important to understand in evaluation of CGM implementation. We selected 20 factors because we planned to develop a survey item for each factor and felt this was a number that would offer comprehensive assessment of PCP perspectives without excessive respondent burden. An expert panel of five VA front-line clinicians (PCPs, clinical pharmacists, and an RN) also participated in multi-voting. The top 20 factors originated from the Innovation, Individuals, and Inner Setting domains, and were selected for assessment via survey.

We drafted two candidate items for each factor and asked the research team to vote on each item’s clarity and relevance. Majority vote determined which candidate item was included in the survey. We then grouped the final 20 items into multi-item scales for analysis, based on the item topics: Clinical Benefits of CGM (Innovation domain), PCP Workload Capacity (Inner Setting domain), PCP Knowledge about CGM (Individuals domain), Access to Resources for CGM (Inner Setting domain) and Support from Leadership & Other Services (Inner Setting domain), and All responses used a 5-point Likert Scale (1-Strongly Disagree to 5-Strongly Agree).

We added 4 items about demographic and practice characteristics. Along with the item used to measure the PCP-level outcome, the final survey involved 32 items that included 19 items across 5 scales (Appendix). Survey instructions indicated that the questions were about CGM for insulin-treated patients with type 2 diabetes.

### Survey sample and administration

All PCPs in VA were eligible [physicians (MD, DO), advanced practice registered nurses (APRN) and physician assistants (PA)] and were identified using the VA Corporate Data Warehouse. Each PCP received an emailed personalized survey link via Qualtrics [27]. There were 7,682 PCPs who received the survey invitation, across two waves between October 2023 and April 2024, with two reminders each sent at 3–4-day intervals. Participation in the survey was voluntary and responses were confidential; items could be skipped.

### Outcome measures

#### PCP intent to discuss CGM

We used the survey item: “I am likely to initiate discussions about starting CGM in the next 3 months with eligible patients who have type 2 diabetes”, with responses on the same 5-point Likert Scale as the other survey items.

### Facility-level uptake outcome

We collected information about facility-level CGM uptake from the VA Corporate Data Warehouse. In July 2023, VA changed eligibility criteria for CGM use to include all insulin-treated patients. Thus, CGM-eligible patients were defined as those aged 18 years and older with type 2 diabetes (as defined by the presence of 1 + outpatient or inpatient ICD-10 code E11.*) on insulin who had at least one primary care visit (as defined by clinic stop code) between August 1, 2023 and March 30, 2024. We did not include patients with type 1 diabetes in the denominator as they are nearly always managed in endocrine specialty care. Facility-level CGM uptake was calculated as the proportion of eligible patients at each site who received a prescription for a CGM sensor in the 8-month period. The start of this timeframe was one month after the expansion of VA’s CGM eligibility criteria.

### Analysis

We calculated 5 scale scores, one for each survey domain, by taking the mean of the individual items for each domain. We then generated descriptive statistics for all variables that included means and standard deviations for continuous variables and frequencies/proportions for categorical variables, as well as percent top box (“Agree” + “Strongly Agree”) for each item, and percent ceiling (scale score ≥ 4) for each scale. Data were normally distributed. We examined bivariate associations between each scale and each outcome variable using Pearson’s correlations. Finally, we examined adjusted associations among the scale scores and each outcome using multivariable regression analyses, with all scales entered simultaneously along with clinician’s self-reported demographic and practice characteristics (gender, years in service, part-time status, and facility type). We identified no concerns about multicollinearity among the scale scores. Analyses of the outcome of intent to discuss CGM were conducted at the PCP level. For analyses of facility-level uptake, all variables were aggregated at the facility level (e.g., mean scale scores across all PCP respondents at a given facility), and only facilities with survey data from at least five PCPs were included in analyses. Missing PCP characteristic data was imputed for analyses; missing values were set to the most frequent response category for that variable. Sensitivity analyses were performed including only clinicians with complete data.

## Results

**Fig. 1:**
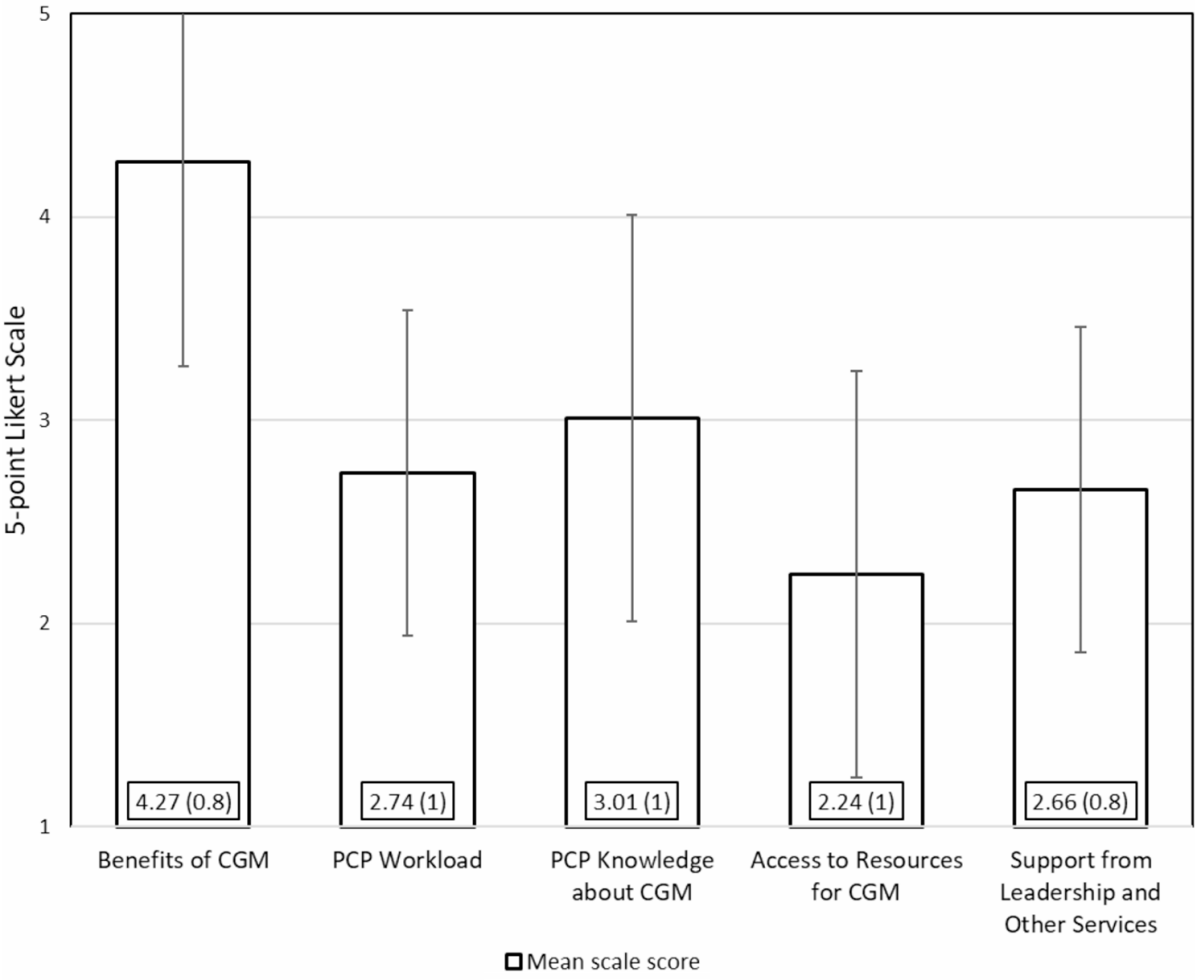
Mean scale scores from PCP survey about CGM implementation

There were 1373 PCPs respondents from 122 VA facilities, representing an 18% response rate. Of these respondents, 59% were female, 62% had been in practice for 11 or more years, 62% had 5 or more half-day clinic sessions per week, and 39% practiced at a VA medical center (versus a community-based outpatient clinic or other setting) (Table 1). Respondents were younger than non-respondents (50 years vs. 52 years, *p* <.0001), had been in VA for fewer years (9 years vs. 11 years, *p* <.0001) and were more often female (67% vs. 62%, *p* =.0021). Scales showed reasonable internal consistency; Cronbach’s alphas for scales ranged from 0.74 to 0.91, values which are above conventional standards of > 0.70. Mean scale scores with standard deviation are in Fig. 1. The percent of PCPs at ceiling was highest for Benefits of CGM (79%), This percent ceiling was much lower for the other scales: PCP Knowledge about CGM (20%), PCP Workload Capacity (15%), Access to Resources for CGM (8%), and Support for Leadership & Other Services (5%).

**Table 1:** Respondent characteristics

Respondent characteristics (*n* = 1373)	*N* (%)
Gender *	
Female	816 (59.4)
Male	393 (28.6)
Another gender	6 (0.4)
Missing	158 (11.5)
**Practice Setting**	
VA Medical Center	537 (39.1)
Community-Based Outpatient Clinic	572 (41.7)
Home-Based Primary Care	111 (8.1)
Other	31 (2.3)
Missing	122 (8.9)
**Years in Clinical Practice**	
Less than 5	191 (13.9)
6 to 10	231 (16.8)
11 to 15	210 (15.3)
16 to 20	178 (13.0)
21+	467 (34.0)
Missing	96 (7.0)
**Number of clinic sessions per week**	
5 or more	857 (62.4)
Less than 5	315 (22.9)
Missing	201 (14.6)

* Response category “Prefer not to answer” was coded as missing

### PCP intent to discuss CGM

**Fig. 2:**
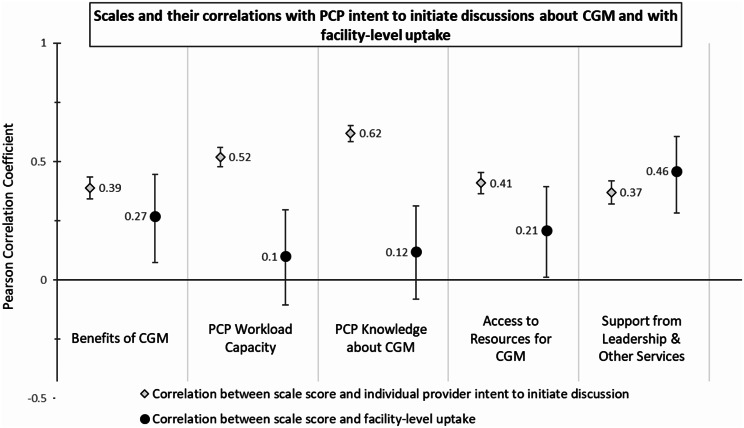
Scales and correlations with PCP intent to initiate discussions about CGM and with facility-level uptake

About half (52%) of respondents agreed or strongly agreed with “I am likely to initiate discussions about starting CGM in the next 3 months with eligible patients who have type 2 diabetes” (mean score 3.36, SD 1.25). In bivariate analyses, all 5 scale scores were positively associated with PCP intent (Benefits of CGM: *r* =.39, *P* <.001; PCP Workload Capacity: *r* =.52, *P* <.001; PCP Knowledge about CGM: *r* =.62, *P* <.001; Access to Resources for CGM: *r* =.41, *P* <.001; Support from Leadership & Other Services: *r* =.37, *P* <.001) (rs > 0.37, ps < 0.001; Fig. 2). All scales except Access to Resources for CGM remained significant predictors of PCP intent to discuss CGM in multivariable analyses controlling for the other scale scores and clinician characteristics (Table 2). PCP Knowledge about CGM was the domain most strongly associated with PCP intent to discuss in both unadjusted (*r* =.62, *P* <.001) and adjusted (B = 0.54, *P* <.001) analyses. The multivariable analyses also revealed that clinicians reported greater intent if they worked at a VA medical center v. any other practice setting (B = 0.14, *P* =.009), and lesser uptake if they had 11 or more years in practice v. 10 years or less (B = -0.18, *P* <.001).

**Table 2:** Multivariable linear regression analysis: determinants of PCP intent to discuss and facility uptake of CGM

	PCP uptake *^, †^ (*n* = 1204)			Facility uptake ^‡,§^ (*n* = 97)	
**Scale**	**B**	***P*** **-value**		**B**	***P*** **-value**
Benefits of CGM	0.25	< 0.001		0.10	0.03
PCP Workload Capacity	0.27	< 0.001		-0.04	0.40
PCP Knowledge about CGM	0.54	< 0.001		-0.009	0.84
Access to Resources for CGM	0.001	0.98		-0.02	0.55
Support from Leadership & Other Services	0.17	< 0.001		0.18	< 0.001
**Covariates**					
Female	0.03	0.63		0.06	0.39
Years in Clinical Practice > 10	-0.18	< 0.001		0.07	0.33
Less than 5 Clinical Sessions per Week	-0.02	0.74		-0.08	0.31
Care delivered in a VAMC	0.14	0.09		0.002	0.98
Intercept	-0.42	0.02		-0.47	0.03
F Value	128.34	< 0.001		3.70	< 0.001
R^2^	0.49			0.29	

* PCP uptake measured by survey question “I am likely to initiate discussions about starting CGM in the next 3 months with eligible patients who have type 2 diabetes,” on a 5-point Strongly Disagree to Strongly Agree scale

^†^ Model for PCP uptake was at respondent level and included all scales scores and clinician gender, years in service, part-time status, and facility type

^‡^ Facility uptake measured by the proportion of patients aged 18 + with type 2 diabetes on insulin who had a PCP visit and received a prescription for a CGM sensor between August 1, 2023 and March 30, 2024

^§^ Model for facility-level CGM uptake was at facility level and included all scale scores and clinician gender, years in service, part-time status, and facility type

### Facility uptake of CGM

97 facilities had survey data from at least 5 clinicians and were included in analyses on facility-level uptake. Facility-level CGM uptake for patients with type 2 diabetes on insulin ranged from 4.2 to 59.3% (mean 30.7%, median 30.5%). In bivariate analyses, Benefits of CGM (*r* =.27, *P* =.006), Access to Resources for CGM (*r* =.21, *P* =.036), and Support from Leadership & Other Services (*r* =.46, *P* <.001) were positively correlated with facility-level uptake (Fig. 2). There was no significant correlation with PCP Knowledge about CGM or PCP Workload Capacity. In multivariable analyses controlling for the other scale scores and clinician characteristics, only Benefits of CGM (B = 0.10, *P* =.026) and Support from Leadership & Other Services (B = 0.18, *P* <.001) remained significant (Table 2). Support from Leadership & Other Services was the domain most strongly associated with facility-level CGM uptake in both unadjusted and adjusted analyses.

Sensitivity analyses were performed including only clinicians with complete data and revealed no impact on the pattern or significance of results.

## Discussion

We conducted a national survey of VA PCPs to understand their perspectives on CGM implementation in primary care, and how those perspectives affect their intent to initiate CGM discussions with eligible patients and relate to CGM use at the facility level. This is the largest study of which we are aware that examines either of these relationships. PCPs perceive that CGM use offers improved clinical benefits to patients, which is consistent with prior smaller surveys [23, 28, 29]. Despite PCP optimism about CGM, we found that several enablers of implementation were lacking, including knowledge about CGM, the ability to absorb CGM into existing workload, and the infrastructure to support CGM implementation. We also found different drivers of PCP intent and facility-level CGM uptake. While PCP knowledge had the strongest association with PCP intent to discuss CGM with eligible patients, support from leadership and other services had the strongest association with CGM uptake at the facility level.

PCPs serve as a gateway to patients’ use of health care technology [30, 31] and intent to discuss CGM use with eligible patients reflects how they experience that position. Intent to discuss was most strongly associated with PCP knowledge, but only 20% of PCPs agreed their knowledge was adequate. Inadequate clinician knowledge about CGM has been reported in other studies [20, 21, 24, 28]; our study adds to the literature by demonstrating a strong negative impact of these shortcomings on PCPs’ planned behavior. Thus, PCP education should be a high-priority target for supporting CGM implementation. Primary care physicians and APRNs tend to rank conferences and meetings as most effective for learning about CGM, followed by websites, training modules, or other online resources [21, 24]. Studies are needed to determine the most acceptable, feasible, efficient, and effective strategies for PCP education about CGM use.

In addition to PCP knowledge, we found other factors were associated with intent to discuss, though less strongly so: the capacity to absorb CGM into existing PCP workload and the infrastructure (resources and other services) to support CGM use. Prior research has identified specific processes that cause difficulty and are related to both workload and infrastructure, such as insufficient time to review CGM data and insufficient time to discuss CGM data with patients [23, 28, 32]. Time constraints are a consistent feature of primary care work in general, thus it is important to consider how workflows may be optimized to support innovations. A common infrastructure challenge is the lack of efficient processes to incorporate CGM data into EHRs [22, 23, 33]. In a study examining how PCPs engage with CGM data, a small national survey found that the majority of PCP respondents either manually entered CGM data into a visit note (35%) or uploaded a scanned image of CGM data into the EHR (48%). Only 15% of surveyed clinicians reported that CGM data reports were integrated with their EHR [23]. Lack of integration with the EHR is a challenge echoed in systematic reviews of remote patient monitoring, the broad category of biosensing technology into which CGM falls [34, 35]. 

There is wide variation in facility uptake of CGM and some facilities are providing CGM at higher rates to eligible patients with type 2 diabetes. Our survey sheds light on that variation, finding that the strongest predictor of facility-level uptake was involvement of other services. The scale measuring support from other services asked about support from leadership, Nursing, Pharmacy, Endocrinology, and other clinical staff. In contrast to the findings for PCP intent to discuss CGM, facility uptake was not associated with PCP knowledge and PCP workload capacity. These findings suggest that interprofessional and multidisciplinary support for CGM is a promising target that addresses PCP concerns about workload and resources through sharing clinical responsibilities among other qualified staff. A scoping review of implementation of remote patient monitoring found that, among clinicians, NPs and physicians are the main groups that are responsible for the core components [36]. While some NPs practice as PCPs, others function in different roles outside of primary care but could support CGM. One study found that nurses would be more likely to prescribe CGM if they could engage in e-consults with endocrinologists, had access to telementoring sessions with a specialty team, or had the ability to refer patients to a specialty care center when patients needed additional information or clinical support [21]. Clinical pharmacists are also well-positioned to assist with CGM implementation [28, 37, 38]. A systematic review found that pharmacist-driven CGM implementation can improve patient empowerment, quality of life, and clinical outcomes, but that educational, logistical, workflow, and financial barriers should be assessed and addressed [28]. This is a particularly promising approach in the VA, where under federal law clinical pharmacists’ scope includes independent management of diabetes and CGM without direct supervision of a physician. Community health workers may also help to support CGM management. These individuals can support patient engagement with health care technology, and have the benefit of understanding challenges facing patients of similar backgrounds, as well as the training and ability to overcome language barriers [39]. The best interprofessional and multidisciplinary approach for a given site will depend on local staffing and training, and lessons may be learned from studying those facilities with higher uptake.

### Limitations

Limitations of this study include the potential for non-response bias. Respondents were a little younger and more often female; whether these characteristics also reflect attitudes towards CGM in general should be explored in future work. The response rate was 18%, though this, the response rate is similar to that of other VA clinician surveys [40, 41]. The study was conducted solely within the VA health system, which may arrange and deliver care differently than other health care settings. However, as described above, many of our findings are aligned with those from previous literature. VA primary care staffing, organized under a patient-centered medical home model, is quite robust, such that facility factors may play an even greater role outside VA. Due to variation in facility-level policy, it is possible that some PCPs were prescribing CGM prior to the expanded policy of July 2023; this would increase the heterogeneity of the population in terms of knowledge and intent to initiate discussions about CGM. We did not assess concerns about reimbursement since all insulin-treated patients with type 2 diabetes in VA are eligible for CGM, and reimbursement is not a barrier to use.

## Conclusions

PCPs believe that CGM use can improve the care of eligible patients with type 2 diabetes, but there are clinician- and system-level barriers to implementation in primary care. Professional training for PCPs about CGM use may increase the likelihood they offer it to eligible patients and support from other clinicians such as clinical pharmacists and nurses can facilitate prescribing and patient use. Further work is needed to identify the most promising workflows and processes to improve CGM use in primary care practices.

## Electronic supplementary material

Below is the link to the electronic supplementary material.

Supplementary Material 1

## Data Availability

Data is provided within the manuscript. The original dataset may be available upon reasonable request.

## Declarations

### Disclaimer

The research presented in this article is not intended to reflect the views of the Department of Veterans Affairs or the United States government.
