# Supplementary material for: Determinants of implementation of continuous glucose monitoring for patients with Insulin-Treated type 2 diabetes: a national survey of primary care providers
Source: BMC Prim Care. 2025 Mar 8;26:68. doi: 10.1186/s12875-025-02764-7 (PMC11889852; doi:10.1186/s12875-025-02764-7)

**Appendix** – Survey of Primary Care Providers on Implementation of Continuous Glucose Monitoring for Patients with Type 2 Diabetes on Insulin in Primary Care


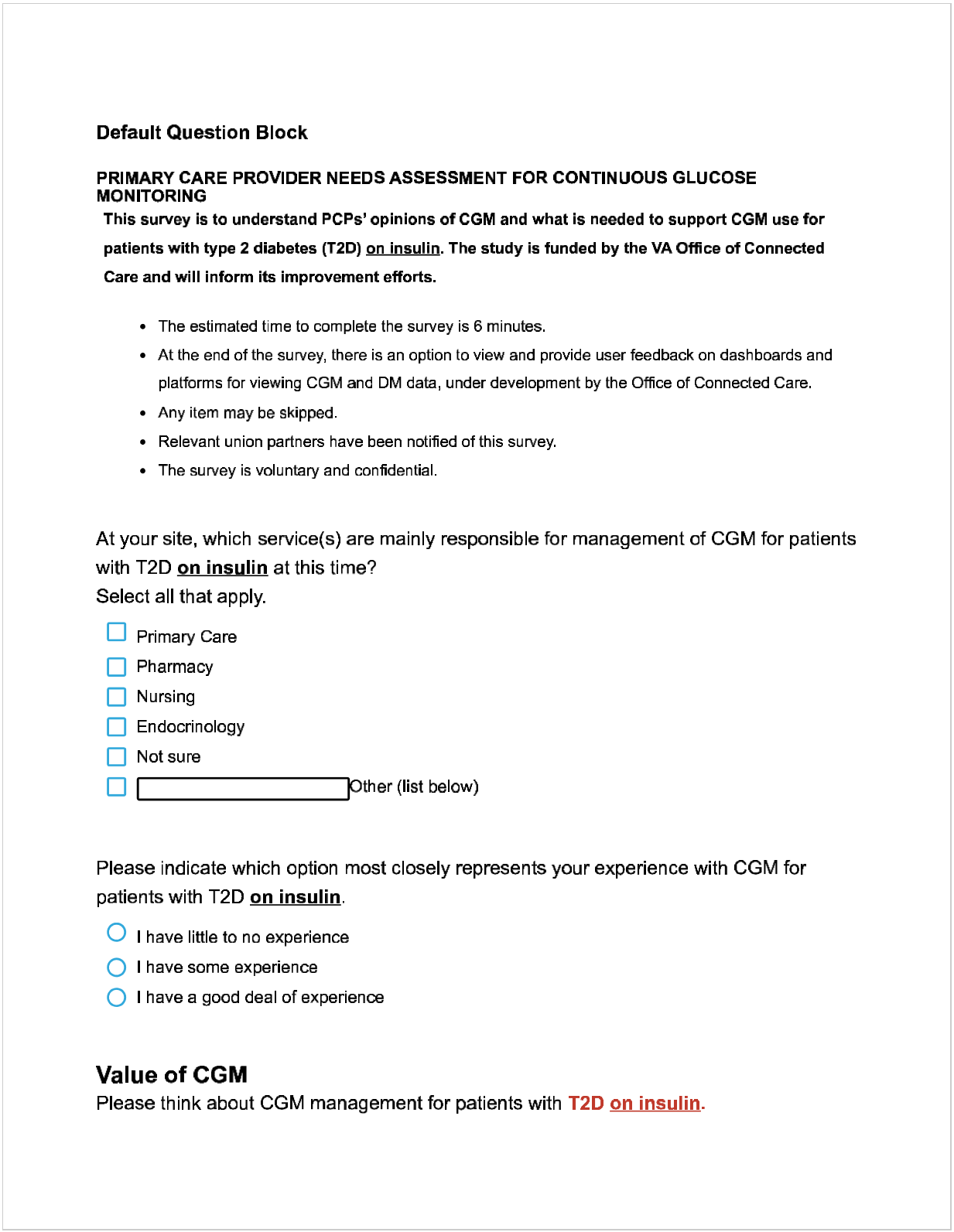


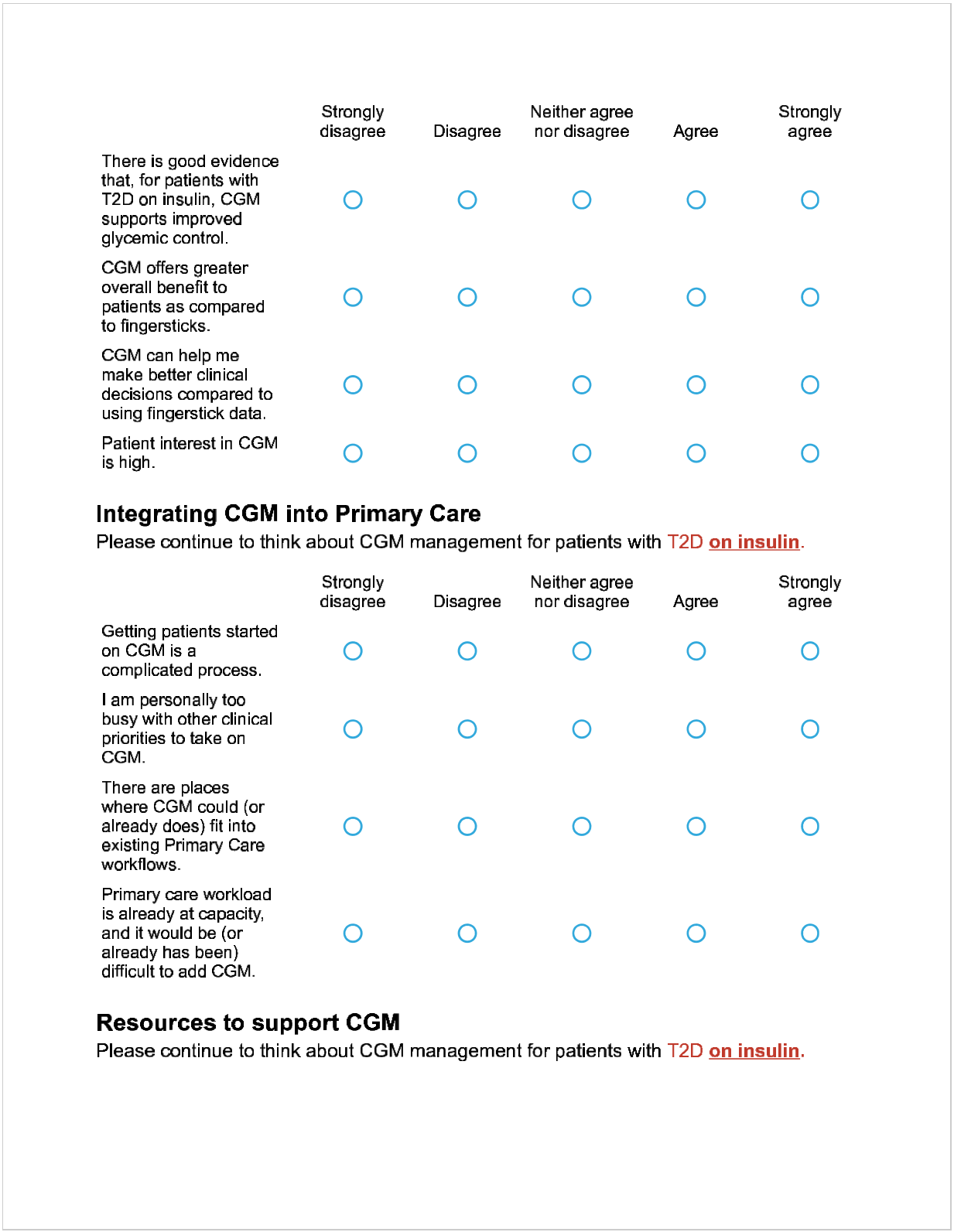


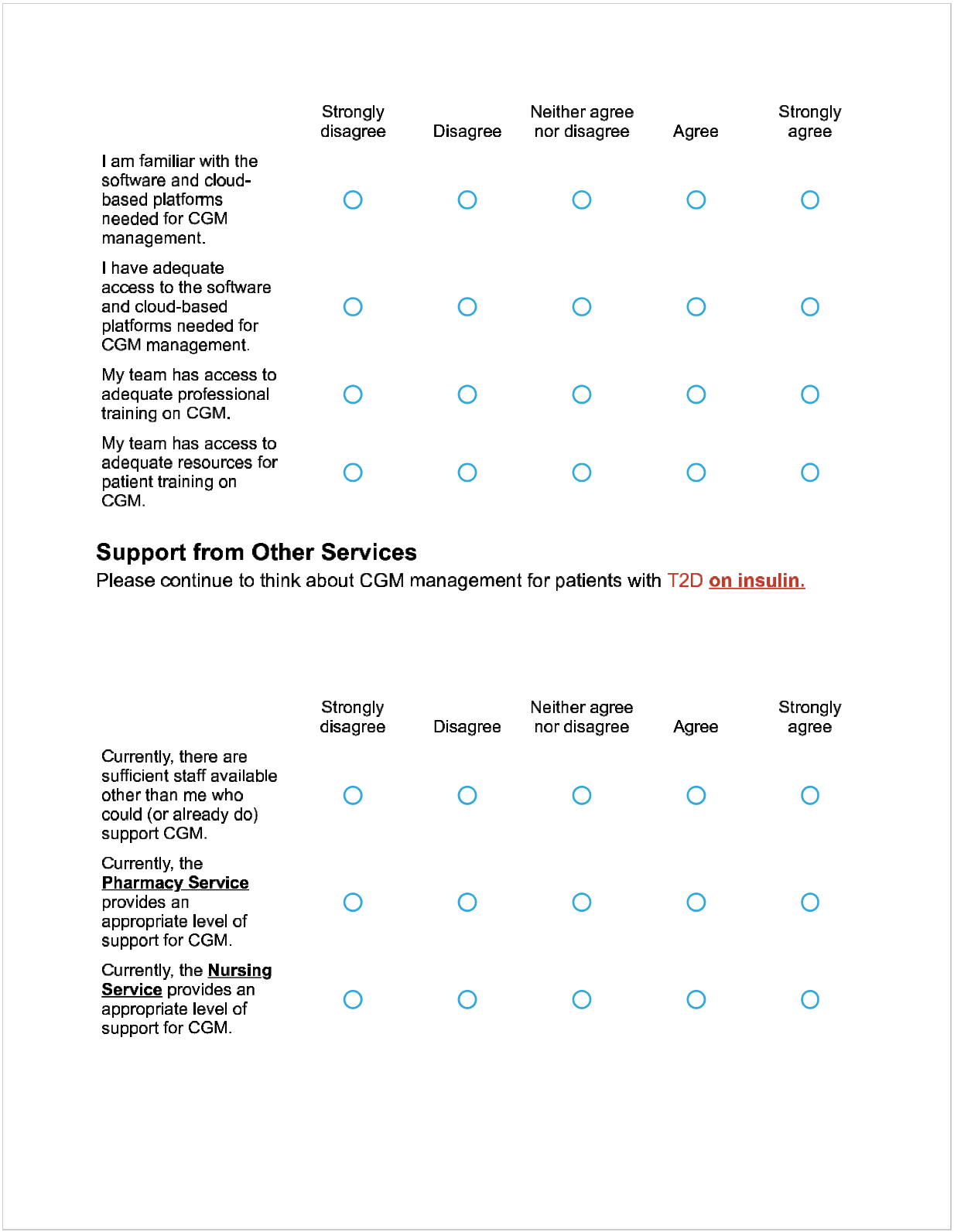


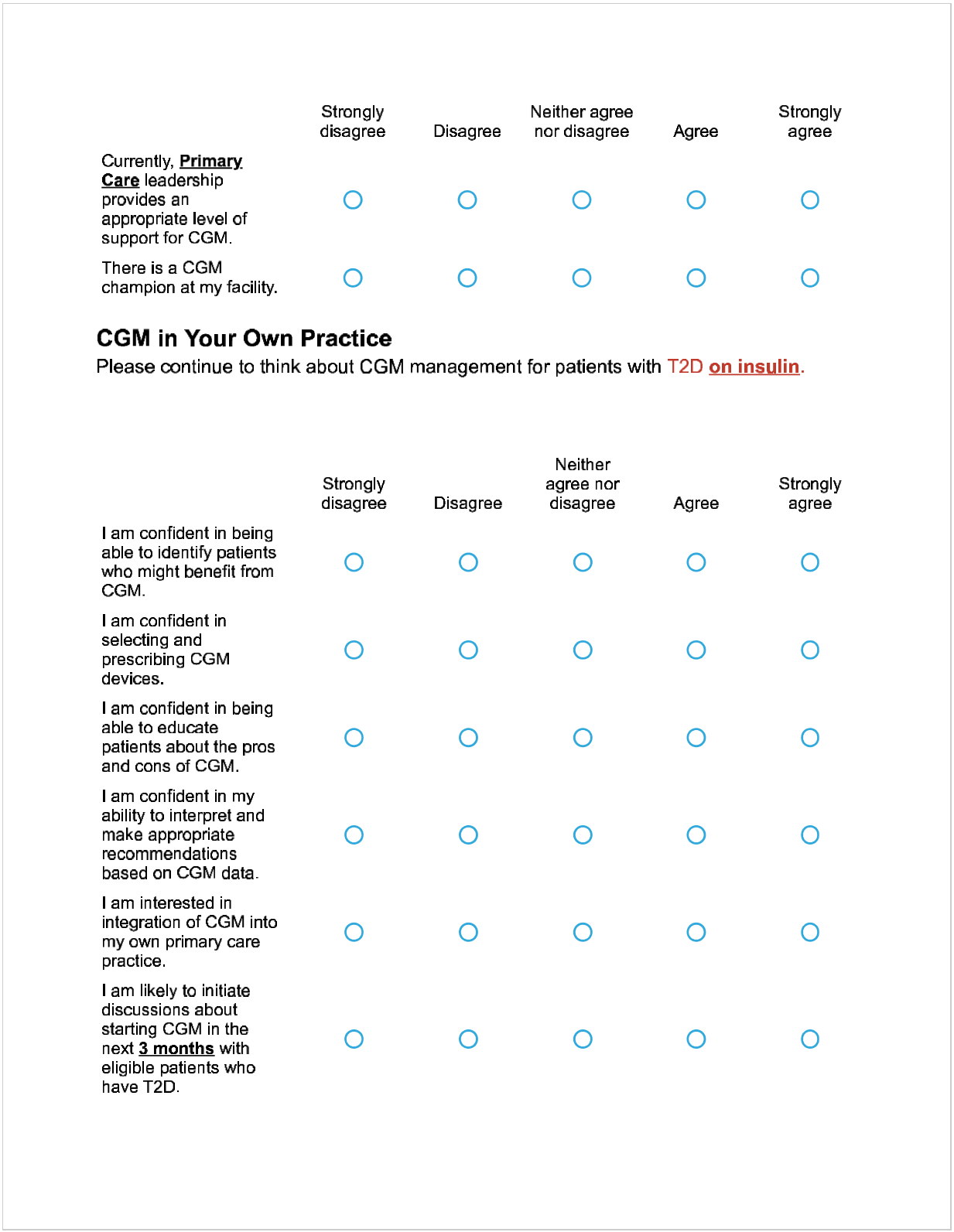


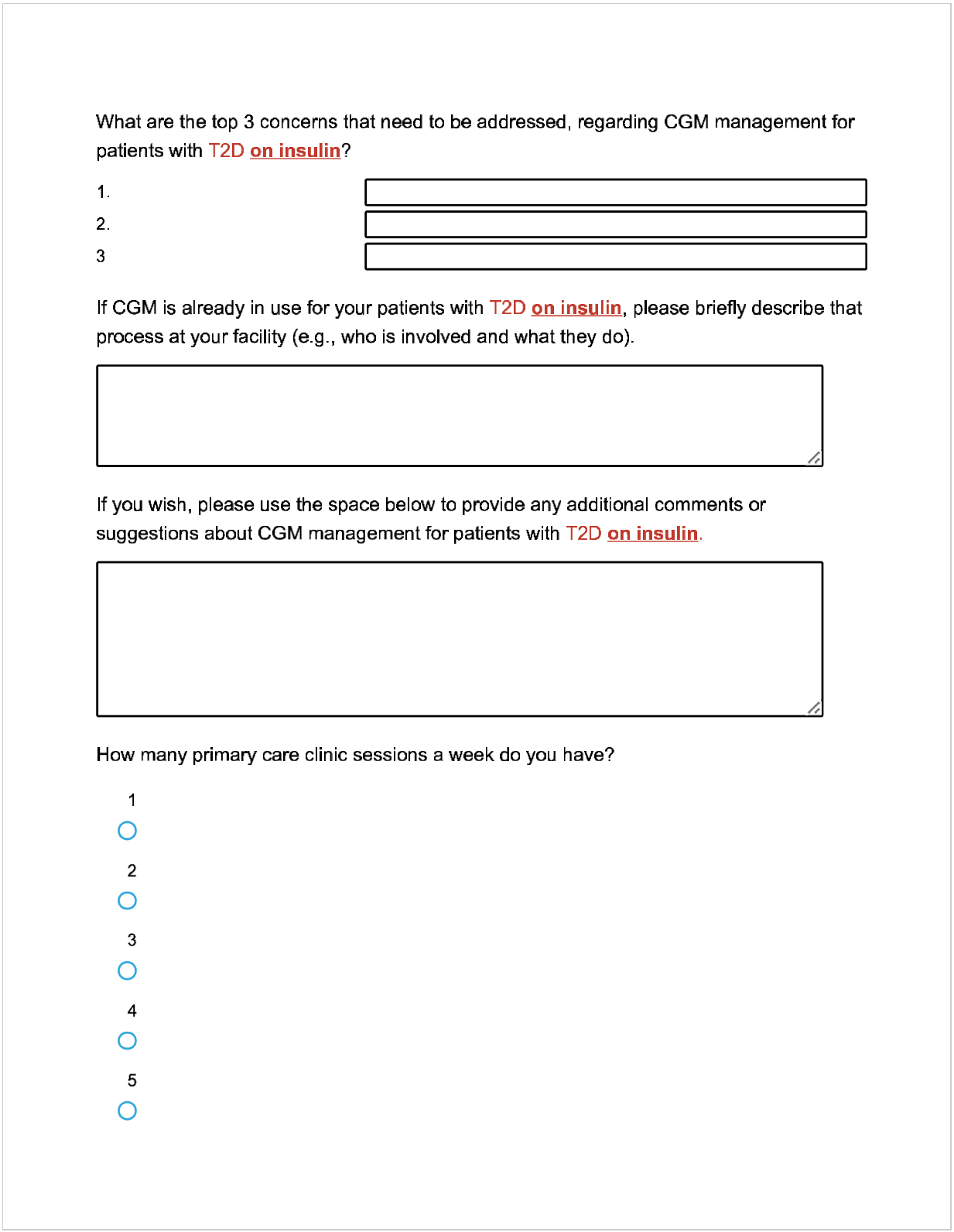


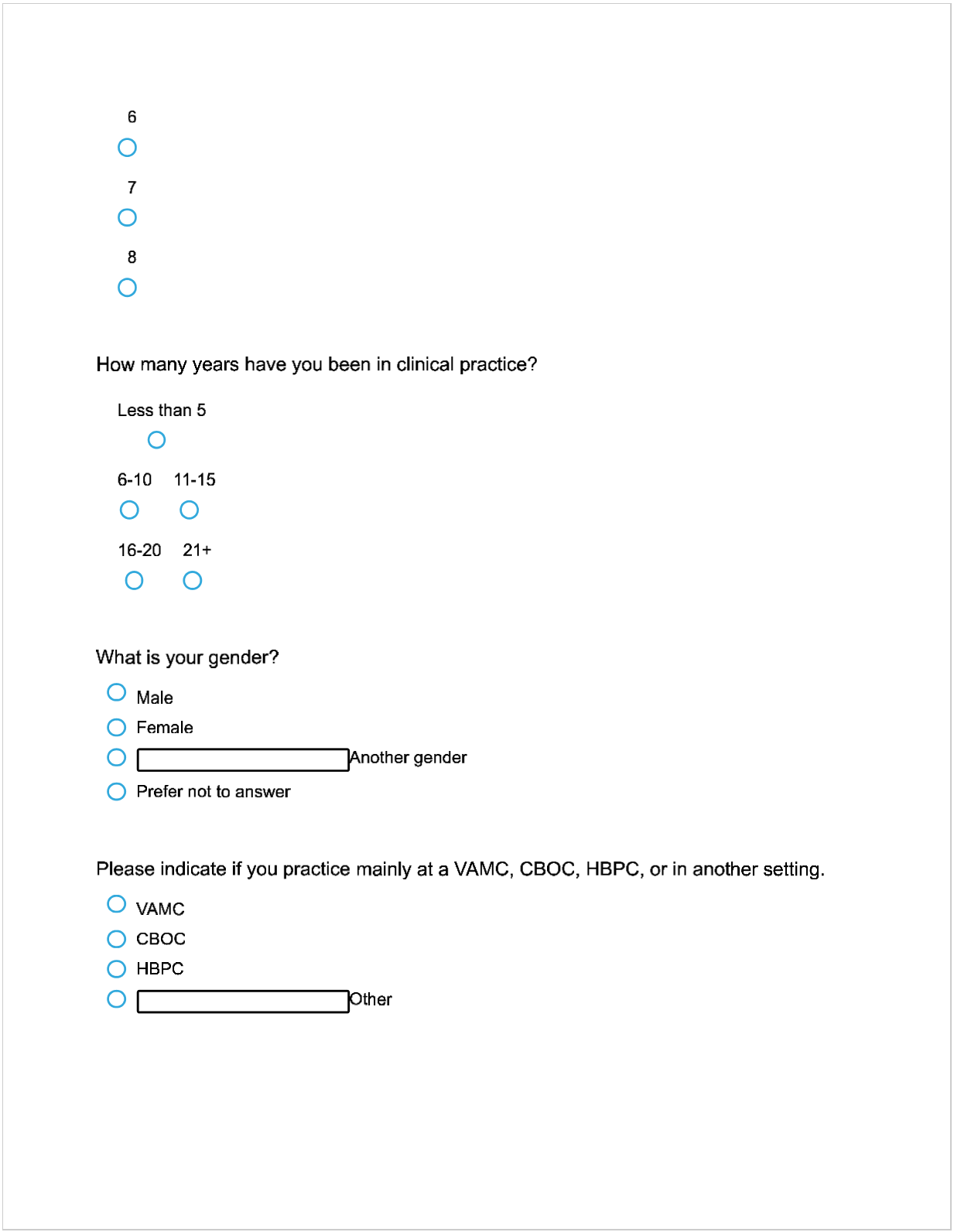

Supplement: Supplementary file 1 — Supplementary Material 1 [file 12875_2025_2764_MOESM1_ESM.docx]
